# Negative impacts of insecticide‐treated methods and slot traps for trapping of *Ips cembrae* on nontarget invertebrates

**DOI:** 10.1002/ps.8716

**Published:** 2025-02-13

**Authors:** Špoula Jakub, Foit Jiří, Resnerová Karolina, Kula Emanuel

**Affiliations:** ^1^ Department of Forest Protection and Wildlife Management Mendel University in Brno, Faculty of Forestry and Wood Technology Brno Czech Republic; ^2^ Forestry & Game Management Research Institute Jíloviště Czech Republic; ^3^ Faculty of Forestry and Wood Sciences, Department of Forest Protection and Entomology Czech University of Life Sciences Prague Prague Czech Republic

**Keywords:** Cembräwit, cypermethrin, *Larix*, saproxylic beetles, insecticide‐treated traps, mass trapping

## Abstract

**BACKGROUND:**

*Ips cembrae* is serious forest pest of European larch (*Larix decidua*). The negative impacts on nontarget invertebrates of the use of different *I. cembrae* trapping methods has not yet been evaluated, although mortality of nontarget invertebrates may occur.

**RESULTS:**

Between 2016 and 2022, the impact of different *I. cembrae* trapping methods on nontarget invertebrates was assessed. Four trapping methods (slot traps, insecticide‐treated tripods, logs and trees) baited with the pheromone lure Cembräwit were tested. Based on larval feeding habits, the captured nontarget invertebrates were grouped into four feeding types: predators, phloexylophagous, saproxylophagous and mycetophagous. The results indicate that predators, especially the clerid beetle *Thanasimus formicarius*, were the most negatively affected group of nontarget invertebrates. A negative effect on a number of IUCN Red List species also was shown, particularly on *Corticeus fraxini* (Tenebrionidae). The results show that Cembräwit‐baited slot traps capture not only *I. cembrae*, but also other serious forest pests such as *I. sexdentatus* and *Pityogenes chalcographus*. The overall effect of different trapping methods on *I. cembrae* population densities has not been studied; however, our results indicate that the use of insecticide‐treated methods has a negative effect on *I. cembrae* predators.

**CONCLUSION:**

The negative effect on the predators may be sufficiently large to outweigh any benefits gained from a reduction in *I. cembrae* numbers resulting from trapping. Therefore, in order to reduce the negative impacts on nontarget invertebrates, properly timed and consistent salvage logging should be used rather than insecticide‐treated trapping methods. © 2025 The Author(s). *Pest Management Science* published by John Wiley & Sons Ltd on behalf of Society of Chemical Industry.

## Graphical abstract

The negative impacts of using insecticide‐treated methods and slot traps against *Ips cembrae* were evaluated. Negative impacts on predator species of bark beetles such as *Thanasimus formicarius* and Red‐Listed species were described.

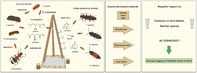

## 1 INTRODUCTION

Bark beetles are common forest pests in Eurasia and America,^1^, ^2^, ^3^ capable of causing severe outbreaks under suitable climatic conditions.^4^, ^5^ Such bark beetle outbreaks are expected to become more frequent with ongoing climate change.^3^ In recent decades, bark beetles from the genera *Ips* and *Dendroctonus* have destroyed millions of hectares of coniferous forests in the Northern Hemisphere,^6^, ^7^ with serious economic consequences.^8^, ^9^ One of the bark beetle species causing local outbreaks^10^, ^11^, ^12^, ^13^ is the large larch bark beetle (*Ips cembrae* Heer), the most important pest of European larch (*Larix decidua* Mill.) in Europe. Various methods have been tested to control populations of *I. cembrae* ^14^, ^15^, ^16^, ^17^ without consideration of their effects on nontarget invertebrates.

Salvage logging is the most effective method to mitigate outbreaks and reduce bark beetle populations.^18^, ^19^, ^20^, ^21^ However, salvage logging often leads to homogenization of habitats and can lead to a decline in biodiversity.^22^ Along with salvage logging, trap trees and slot traps are used to control bark beetle populations.^18^, ^23^ Since 1977, when the aggregation pheromone blend of *Ips typographus* was identified, slot traps (STs), insecticide‐treated trap trees, logs, and tripods baited with species‐specific pheromone lures have been used.^8^ Many studies have been published (e.g.^17^, ^24^, ^25^) testing the effectiveness of different methods baited with different pheromone lures against bark beetles. Some authors have found that tripods and logs are more effective in capturing bark beetles than STs.^26^, ^27^ However, several authors have argued that pheromone‐baited methods are generally ineffective, as the proportion of beetles captured is insignificant compared to the total population.^28^

The use of insecticides is considered to be the most effective and economical method of pest management^29^ and currently pyrethroid insecticides are widely used in crop protection and forestry.^30^ Pyrethroids are synthetic derivatives of natural pyrethrins extracted from *Chrysanthemum cinerariaefolium* (Trevir.) Vis. They exhibit low toxicity to mammals and birds,^31^, ^32^, ^33^ yet high efficacy against many insect pests owing to insects' smaller size, lower body temperature and more sensitive sodium channels.^32^ Pyrethroids are nonpersistent and biodegrade rapidly, thereby reducing the potential for bioaccumulation.^34^ An additional advantage of pyrethroids is their low volatility and polarity, which reduce their movement from the application site through air and soil.^35^ The main disadvantage of pyrethroids is their high potential for bioaccumulation in freshwater invertebrates.^36^ Most pyrethroids occur as isomers, with each isomer having different biological activity and therefore different toxicity. Rivera‐Dávila *et al*.^36^ showed that cypermethrin is the least toxic pyrethroid and recommend its use for forest protection against conifer bark beetles.

Some authors have found that baited insecticide‐treated tripods (TTs) and logs are more effective in capturing bark beetles than STs,^26^, ^27^ but some authors have suggested that the use of baited methods is not effective overall because the proportion of beetles captured is negligible in relation to the total population.^28^ Numerous studies (e.g.^37^, ^38^, ^39^, ^40^, ^41^) have documented nontarget arthropods captured by different trapping methods focused on different bark beetle species, however most studies only publish tables with species names and numbers of captured adults. This study aimed to undertake a complete appraisal of nontarget invertebrates captured by the methods used to trap *I. cembrae*. The specific objective of this study was to define the negative effects on nontarget invertebrates of using baited and insecticide‐treated methods for trapping of *I. cembrae*, with a focus on IUCN Red‐Listed species and bark beetles.

## 2 MATERIALS AND METHODS

### 2.1 Study sites

**Figure 1:**
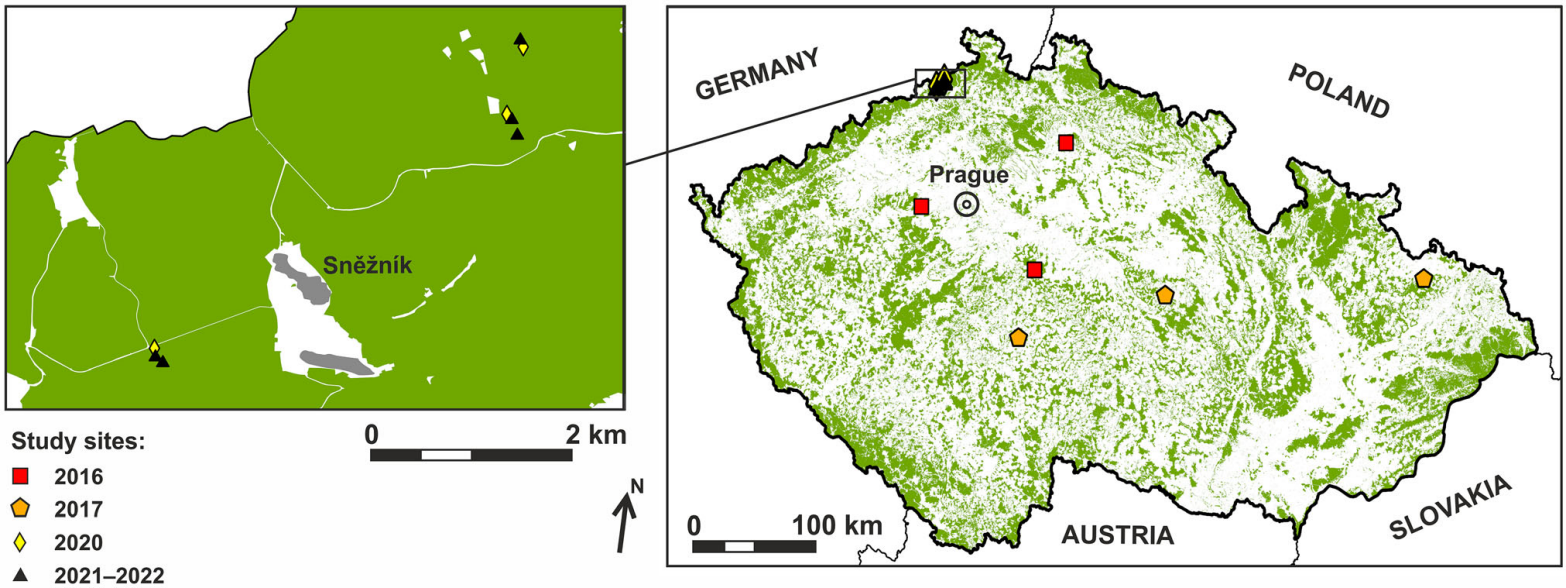
Locations of the study sites. The sites are marked with different symbols representing the years of the study.

The study was conducted in 14 sites within monocultures or mixed stands of *L. decidua*, located throughout the Czech Republic (Fig. 1) at altitudes ranging from 320 to 595 m a.s.l. (Table 1).

**Table 1:** Study site characteristics and number of trapping methods per site

Locality	GPS	Year	Stand age	Altitude (m a.s.l.)	Slot trap	Tripod	Log	Baited tree	Nonbaited tree
Nouzov	50.220 N, 13.944E	2016	40–60	405	5	5			
Stříbrná Skalice	49.919 N, 14.843E	2016	61–80	420	5	5			
Žehrov	50.522 N, 15.091E	2016	61–80	320	5	5			
Babákov	49.803 N,15.892E	2017	81–100	560		4			
Jiřetice	49.591 N, 14.724E	2017	41–60	480	5	5			
Raduň	49.880 N, 17.954E	2017	41–60	400	5	5			
Tisá 1	50.786 N, 14.062E	2020	21–40	595	4	4			
Kristin Hrádek 1	50.820 N, 14.124E	2020	21–40	510	4	4			
Kristin Hrádek 2	50.812 N, 14.1226E	2020	41–60	450	4	4			
Tisá 1	50.786 N, 14.062E	2021–2022	21–40	595	1		1	1	1
Tisá 2	50.786 N, 14.063E	2021–2022	21–40	590	1		1	1	1
Kristin Hrádek 1	50.820 N, 14.124E	2021–2022	21–40	510	1		1	1	1
Kristin Hrádek 2	50.810 N, 14.125E	2021–2022	21–40	510	1		1	1	1
Kristin Hrádek 3	50.812 N, 14.122E	2021–2022	41–60	450	1		1	1	1

### 2.2 Trapping methods

**Figure 2:**
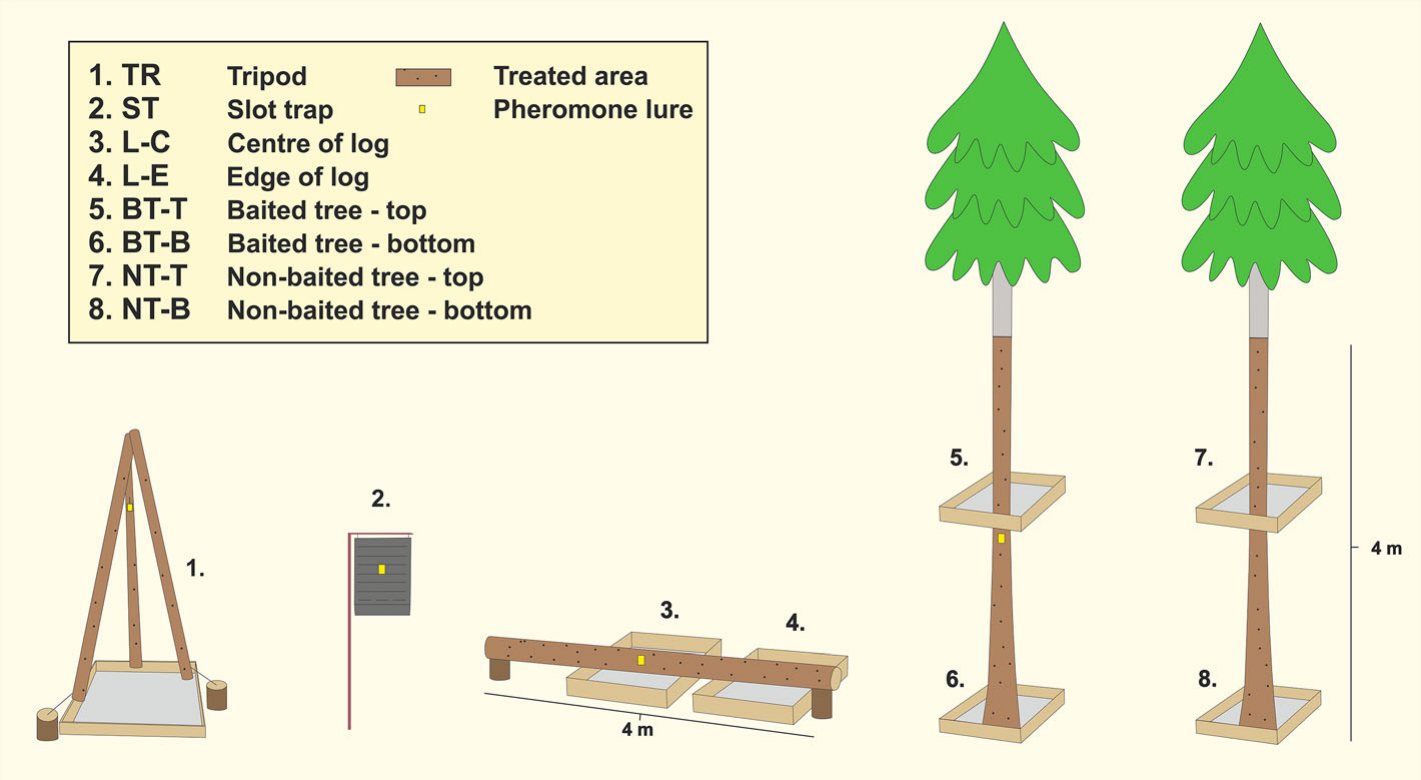
Experimental setup of different *Ips cembrae* trapping methods with position of pheromone lures and frass sheets.

Four trapping methods were used to capture *I. cembrae*: STs, insecticide‐treated tripods (TT), logs and trees. The efficiency and detailed construction of the STs and TTs can be found in other studies,^12^, ^16^ and that of insecticide‐treated logs and insecticide‐treated trees elsewhere.^17^ The logs (L) were divided into two different treatments: centre (L‐C) and edge (L‐E). The trees also were divided into two different treatments: nonbaited (NT) and baited with a pheromone lure (BT). Samples were collected at two points on the trees, the bottom and the top of the tree, resulting in data collected at the bottom of baited (BT‐B) and nonbaited (NT‐B) trees, and the top of baited (BT‐T) and nonbaited (NT‐T) trees. All methods except ST had frass sheets (1 × 1 m) attached to them from which samples were taken (Fig. 2).

Slot traps (Theysohn), tripods and logs, were placed 10–15 m from the larch stand edge and exactly 15 m from other methods. The trees consisted of two healthy trees per stand selected along the stand edge.

Cembräwit (Witasek, Feldkirchen in Kärnten, Austria) pheromone lures were set in early April and replaced in June. In the STs, the lures were placed at a height of ≈1.6 m and on the tripod traps at a height of 1.8 m. On the logs, the lures were placed in the centre of the log (2 m from the edge) and on trees, the lure was placed at a height of 2 m.

The surface of the logs, tripods and trees was treated with insecticide every month from April to September. A 1% solution of Vaztak Active (Agrospol Czech Spol. S.R.O., Pizen, Czech Republic; active substance: alfa−cypermethrin 50 g·L^−1^) was used in 2016, 2017 and 2020 and a 1% solution of Forester (Arysta LifeScience, Cary, NC, USA; active substance: cypermethrin 100 g·L^−1^) was used in 2021 and 2022. Slot traps were not treated with insecticide.

All insects were collected every 10–14 days from the STs, and frass sheets wereplaced under the tripods, logs and the two sampling positions on the trees. The samples were preserved in a 75% ethanol solution for later analysis in the laboratory.

In the laboratory the samples of captured insects were sorted into orders, and the number of individuals in each order was recorded. Subsequently, beetles (order Coleoptera) were identified to species, genus or family level. All saproxylic beetles were identified to species level.

Captured saproxylic beetles were grouped into four feeding types based on their larval feeding habits: (i) predators (predators of saproxylic species), (ii) phloeoxylophagous (feeding on phloem and partially on wood), (iii) saproxylophagous (feeding on partially decomposed wood), and (iv) mycetophagous (feeding on wood‐decomposing fungi). The classification was based on the that of Seibold *et al*.,^42^ adjusted and supplemented according to our observations and additional literature. The number of IUCN Red List species in each sample also was recorded.

### 2.3 Data analysis

As all nontarget insect fauna were recorded at least to order level, data analyses were primarily focused on saproxylic beetles, the most numerous and expectably the most responsive group in the dataset. In the first analysis, the number of nontarget invertebrates captured (response variable) was treated as a count variable, whereas the trapping method was treated as categorical factor. Zero‐inflated generalized linear mixed models (GLMM) with a log‐link function and negative binomial distribution were used to test the effect of *I. cembrae* trapping methods (fixed effect factor) on four feeding categories of nontarget invertebrates and Red List species using the *glmmTMB* function from the glmmtmb package.^43^

In the second analysis, counts of individuals in all samples (response variable) grouped by fixed categorical factor (feeding type) were tested without fixed categorical factor (trapping method) to find, which feeding type was the most affected group using GLMM with negative binomial distribution, using function *glmm.nb* from package lme4.^44^

For the third analysis, the species were grouped into three categorical factor groups according to host tree type (deciduous, coniferous and polyphagous) and numbers of individuals in samples (response variable) were tested by GLMM using the function *glmmTMB* (see above), where their number was treated as a continuous variable and host trees as a categorical fixed effect. Locality and date of sampling were treated as categorical variables and as random effect factors in all GLMMs. The likelihood ratio test was performed using the *anova* function from the stats package. Categories of trapping methods and feeding types were compared with a pairwise *post hoc* test using the function *glht* from the package multicomp.^45^ The data were analyzed in R (v4.4.0) and visualized in rstudio (v2024.04.1 + 748).

A partial canonical correspondence analysis (pCCA) performed in canoco (v5) was used to assess the effects of methods (ST and TR) on bark beetle species composition. The study site and sampling date were used as covariates. A Monte Carlo permutation test with 9999 permutations was used to calculate the significance level. The *multipatt* function from the package indicspecies ^46^ was used to find significant indicator bark beetle species for methods in pCCA. All analyses were performed at a confidence level of *α* = 0.05.

## 3 RESULTS

**Figure 3:**
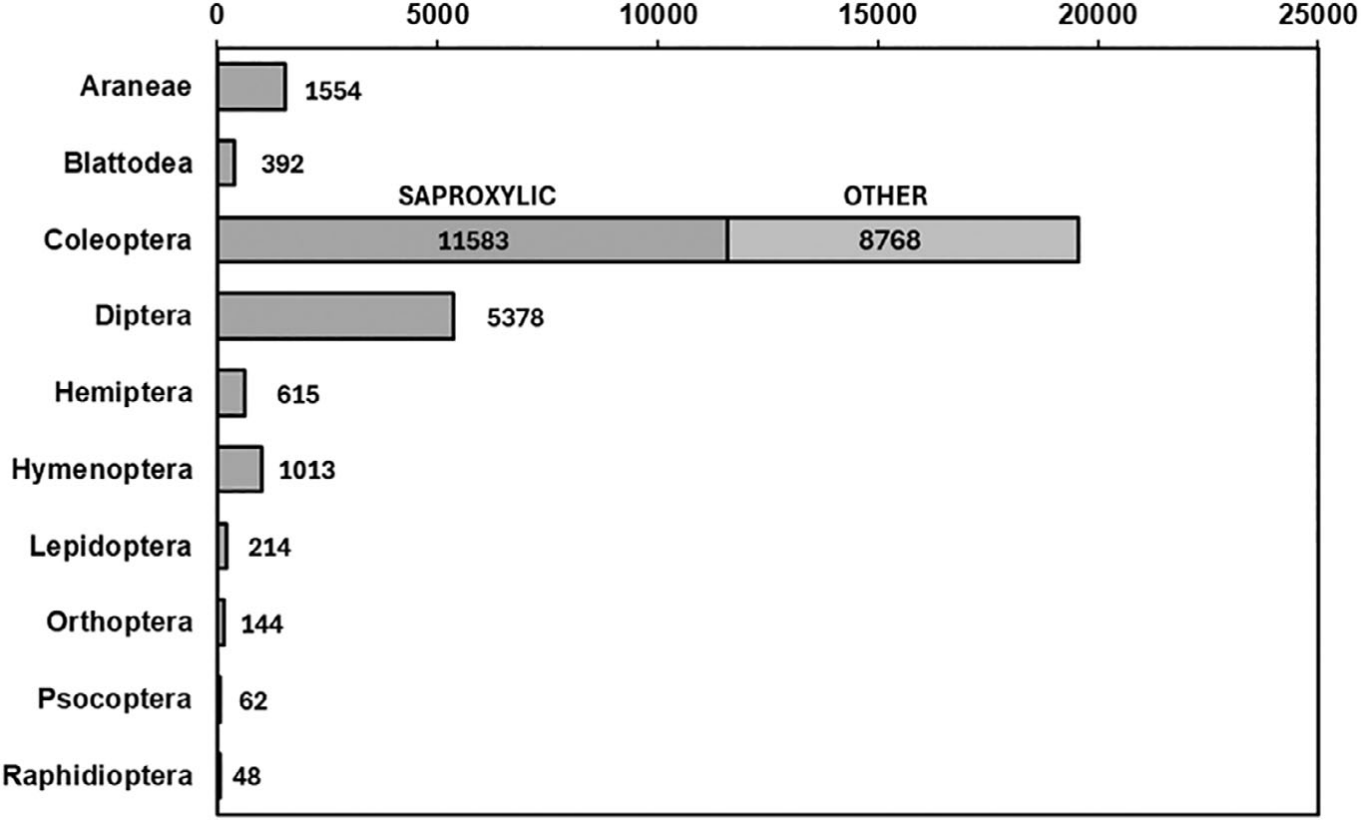
Total number of captured of nontarget invertebrate individuals classified by order across all years and sites.

Between 2016 and 2022, a total of 29 064 nontarget invertebrates were captured across all four trapping methods. Invertebrates of the order Coleoptera were the largest group, representing 67.3% of all nontarget invertebrate individuals captured, followed by Diptera (18.5%) and Araneae (5.4%). The majority of captured beetle individuals (56.6%) were saproxylic (Fig. 3). Orders with <30 captured specimens are not visualized in Fig. 3.

### 3.1 Species composition of the nontarget saproxylic beetles

**Figure 4:**
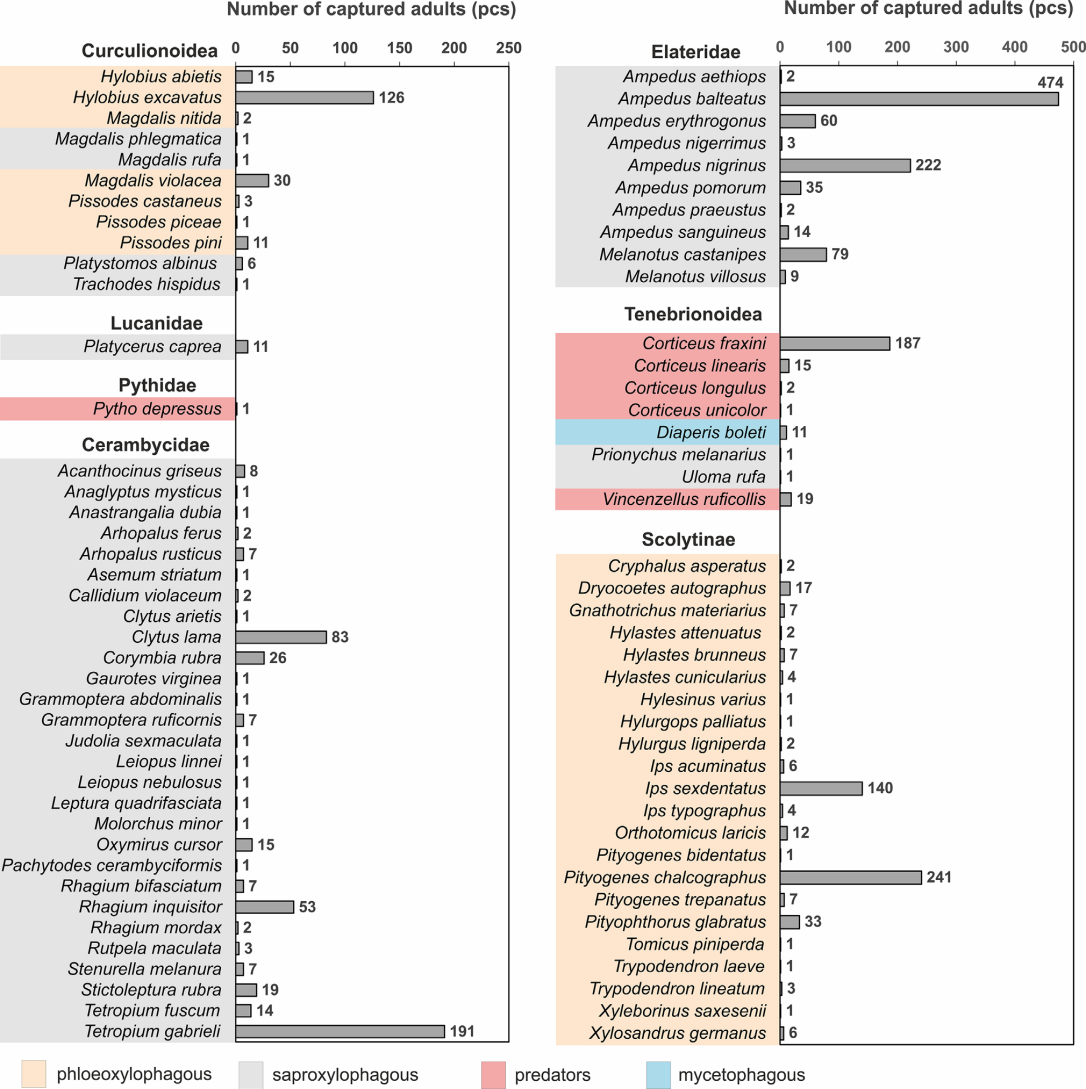
Total number of saproxylic beetle species captured (2020–2022) based on their feeding type.

Saproxylic beetles were the largest group of captured nontarget invertebrates (11 583 individuals) and were represented by 81 species. *Thanasimus formicarius* L. was by far the most numerous species (7289 individuals captured) followed distantly by several other numerous species (*Ampedus balteatus* L., *A. nigrinus* Ger., *Corticeus fraxini* Kug., *Pityogenes chalcographus* L. and *Tetropium gabrieli* Wei.), each consisting of at most several hundred individuals. All representatives of the selected families were determined to species level and are shown in Fig. 4 (except *T. formicarius* for clarity of data).

### 3.2 Feeding types of the nontarget saproxylic beetles

Among nontarget saproxylic beetles, predatory species were by far the most numerous feeding type with 7541 individuals (GLMM: *P* = 0.001, *R* ^2^ = 0.94), of which 96.7% were represented by just one species, *Thanasimus formicarius*. Phloeoxylophagous and saproxylophagous feeding type species were captured less abundantly with 2001 and 1965 individuals recorded, respectively. *Ampedus balteatus* was the most abundant saproxylophagous species with 474 individuals captured, whereas *Pityogenes chalcographus* was the most abundant phloeoxylophagous species with 241 individuals captured. Mycetophagous feeding species, represented by a total of only 76 individuals, were rare in the samples.

**Figure 5:**
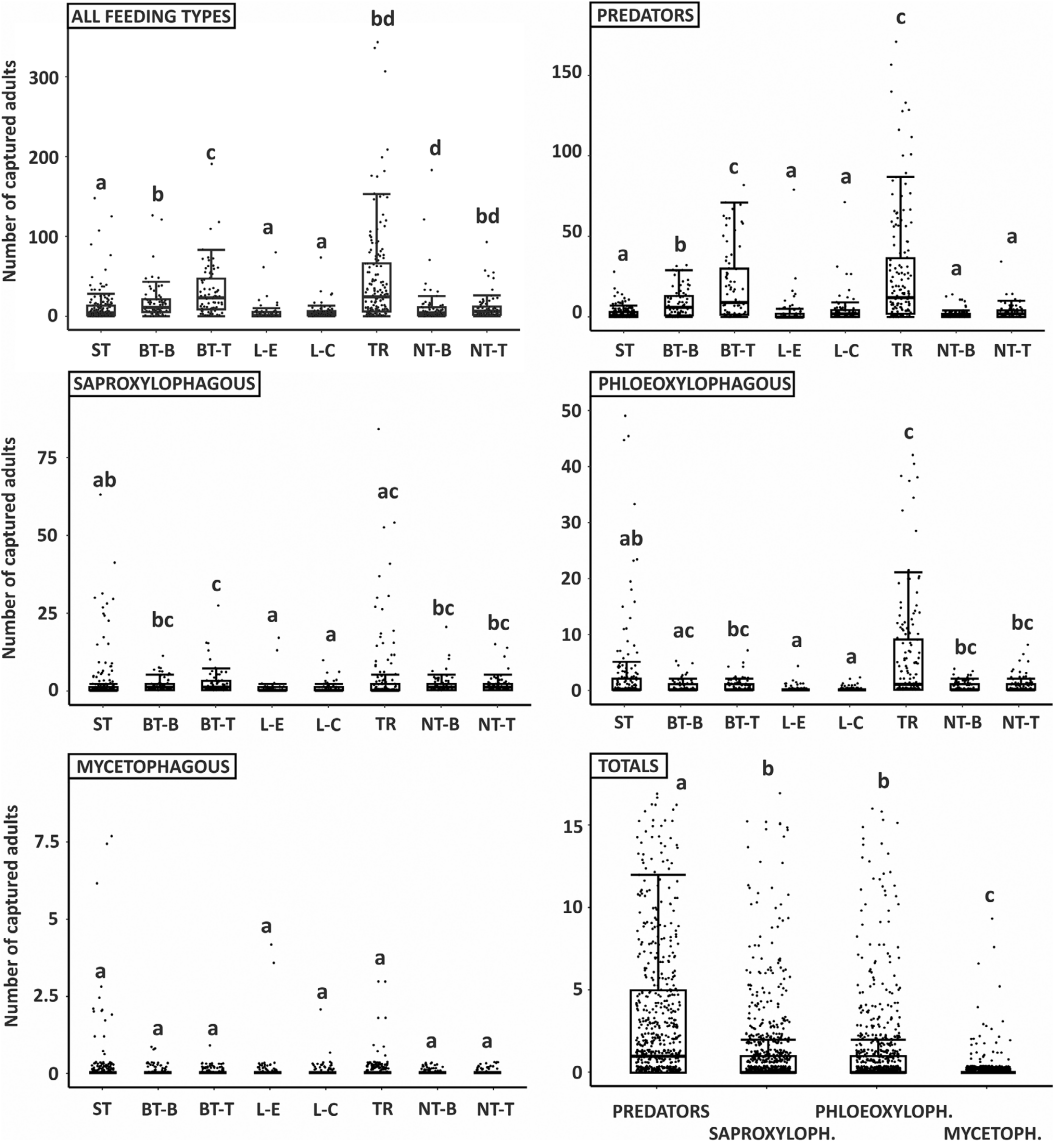
Boxplots showing counts of nontarget saproxylic beetles (in total and separately by feeding type) captured by different trapping methods. Significant differences (*P* < 0.05) are labelled with letters; categories labelled with the same letter did not differ significantly. Lines inside boxes indicate medians, rectangles interquartile range, whiskers minimum and maximum values, and points are jittered samples. ST, slot traps; BT‐B, bottom of baited tree; BT‐T, top of baited tree; L‐E, edge of log; L‐C, center of log; TR, tripod; NT‐B, bottom of nonbaited tree; NT‐T, top of nonbaited tree.

The GLMMs show that trapping method has a highly significant effect on the total number of nontarget saproxylic beetles captured (all feeding types grouped together) (*P* = 0.001, *R* ^2^ = 0.79), as well as on the number of captured individuals of predator (*P* = 0.001, *R* ^2^ = 0.73), saproxylophagous (*P* = 0.001, *R* ^2^ = 0.87), phloeoxylophagous (*P* = 0.001, *R* ^2^ = 0.84) and mycetophagous (*P* = 0.01, *R* ^2^ = 0.93) feeding types of saproxylic beetle (Table 2; Fig. 5). The most distinct effects were found on predator and phloeoxylophagous feeding type groups, where a significantly higher number of individuals was recorded at tripod and, in the case of predators, at the top of baited trees. As predators and phloeoxylophages were the feeding types with the greatest numbers of individuals, a similar pattern also was observed in the total of all captured individuals (all feeding types together) (Table 2; Fig. 5).

**Table 2:** Numbers (mean per sample ± SE) of nontarget saproxylic beetle individuals captured by different trapping methods, in total and split by feeding type

Feeding type	Slot traps	Baited tree, bottom	Baited tree, top	Edge of log	Centre of log	Tripod	Nonbaited tree, bottom	Nonbaited tree, top
All feeding types	10.95 ± 1.61	18.03 ± 2.6	30.97 ± 3.76	5.34 ± 1.54	6.19 ± 1.27	46.61 ± 5.08	11.75 ± 3.13	11.18 ± 1.84
Predator	2.4 ± 3.34	8.5 ± 0.98	19.8 ± 0.39	3.5 ± 0.79	4.8 ± 0.8	27.2 ± 0.34	1.9 ± 2.76	3.4 ± 1.72
Saproxylic	3 ± 0.64	1.5 ± 0.25	2.4 ± 0.5	0.8 ± 0.32	0.8 ± 0.2	5.4 ± 1.54	1.7 ± 0.36	1.7 ± 0.33
Phloeoxylophagous	3.9 ± 0.98	0.6 ± 0.13	0.8 ± 0.15	0.2 ± 0.08	0.2 ± 0.05	7.3 ± 1.12	0.7 ± 0.12	0.9 ± 0.18
Mycetophagous	0.3 ± 0.007	0.04 ± 0.003	0.01 ± 0.002	0.13 ± 0.01	0.04 ± 0.004	0.09 ± 0.003	0	0

### 3.3 Host tree association of the nontarget saproxylic beetles

**Figure 6:**
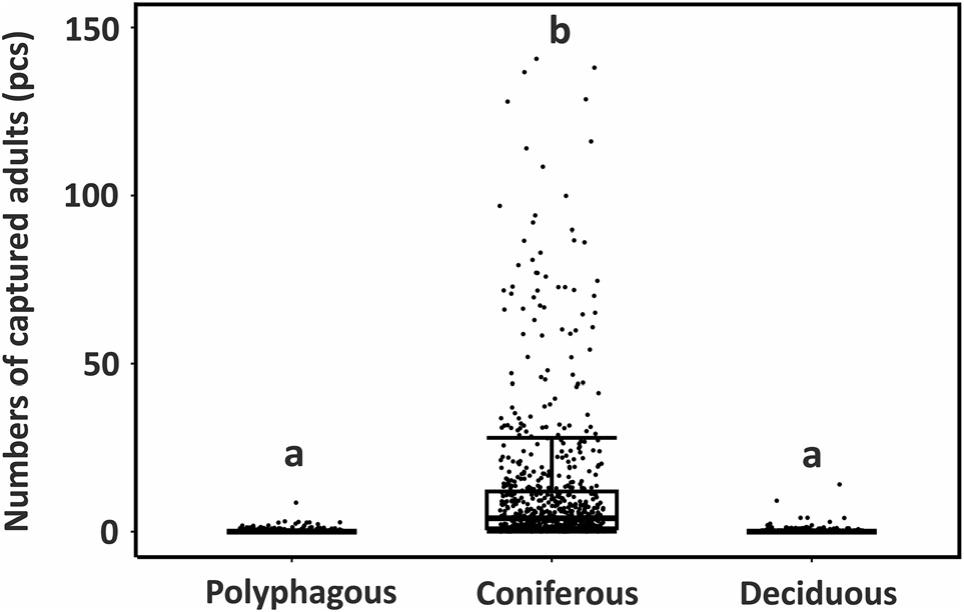
Boxplots of counts of nontarget saproxylic beetles grouped by their association to a host tree species. Significant differences (*P* < 0.05) are labelled with letters, categories labelled with the same letter are not significantly different. Lines inside the boxes indicate medians, rectangles interquartile range, whiskers minimum and maximum values. and points jittered samples.

Nontarget saproxylic beetle species associated with coniferous tree species were predominantly captured (GLMM: *P* = 0.001, *R* ^2^ = 0.86), representing 98.4% of all captured individuals. There was no significant difference between the number of deciduous tree‐associated species and polyphagous species captured (P*p* = 0.75) (Fig. 6).

### 3.4 Red List species of nontarget saproxylic beetles

A total of 207 individuals of IUCN Red List beetles were captured by all trapping methods across all years. Six Red‐Listed tenebrionid species were recorded: *Corticeus fraxini* Kug., *C. linearis* Fab., *C. longulus* Gyll., *C. unicolor* Piller & Mitt., *Prionychus melanarius* Ger. and *Uloma rufa* Piller & Mitt. (Table 3). *Corticeus fraxini*, with 187 captured individuals, was by far the most common species trapped.

**Table 3:** Mean (±SE) number of Red‐Listed saproxylic beetle species captured by different trapping methods

Species	Category^1^	Slot traps	Baited tree, bottom	Baited tree, top	Edge of log	Centre of log	Tripod	Nonbaited tree, bottom	Nonbaited tree, top	Sum
*Corticeus fraxini*	EN	0.3 ± 0.03	0.96 ± 0.15	0.31 ± 0.7	0.29 ± 0.008	0.07 ± 0.36	0.19 ± 0.01	0.19 ± 0.03	0.08 ±	187
*Corticeus linearis*	VU	0.02 ± 0.004	0	0	0	0	0.05 ± 0.02	0.05 ± 0.02	0.07 ± 0.02	15
*Corticeus longulus*	VU	0	0.03 ± 0.005	0	0	0	0	0	0	2
*Corticeus unicolor*	NT	0.01 ± 0.0003	0	0	0	0	0	0	0	1
*Uloma rufa*	VU	0	0	0	0	0.02 ± 0.14	0	0	0	1
*Prionychus melanarius*	EN	0	0	0	0	0	0	0	0.01 ± 0.0003	1
Sum		54	74	23	2	6	36	36	12	

*Note*: 1 Categories of Red List species.

Abbreviations: EN, endangered; VU, vulnerable; NT, near threatened.

**Figure 7:**
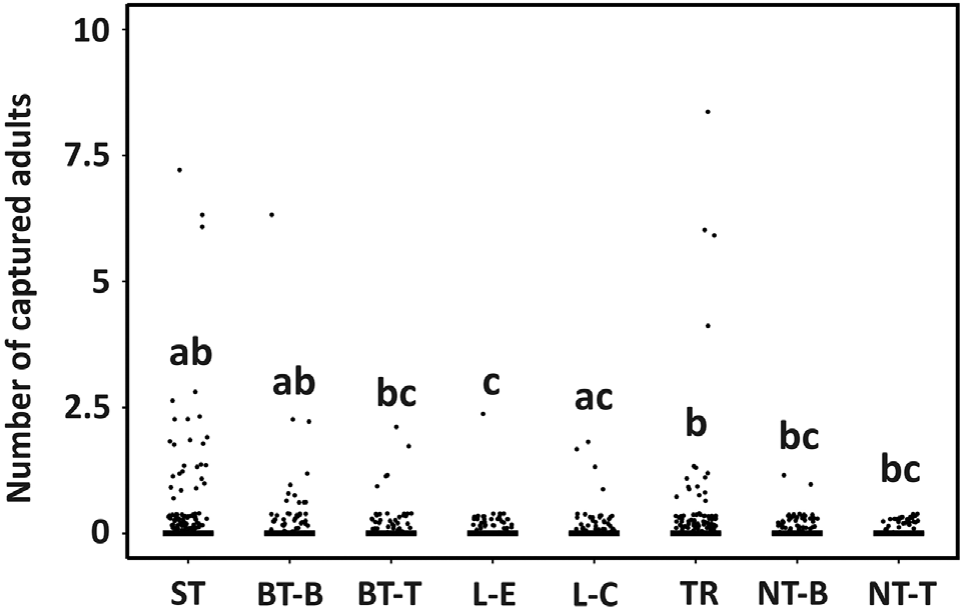
Boxplots showing counts of Red‐Listed nontarget saproxylic beetles captured by different trapping methods. Significant differences (*P* < 0.05) are labelled with letters; categories labelled with the same letter did not differ significantly. Lines indicate medians and points are jittered samples. ST, slot traps; BT‐B, bottom of baited tree; BT‐T, top of baited tree; L‐E, edge of log; L‐C, centre of log; TR, tripod; NT‐B, bottom of nonbaited tree; NT‐T, top of nonbaited tree.

The trapping method had a highly significant effect on the number of red‐listed species captured (GLMM: *P* = 0.001, *R* ^2^ = 0.92). Most individuals were captured at the bottom of baited trees, but the numbers of individuals captured did not significantly differ from other methods, except at the edge of logs (Fig. 7).

### 3.5 Effects on nontarget bark beetles

**Figure 8:**
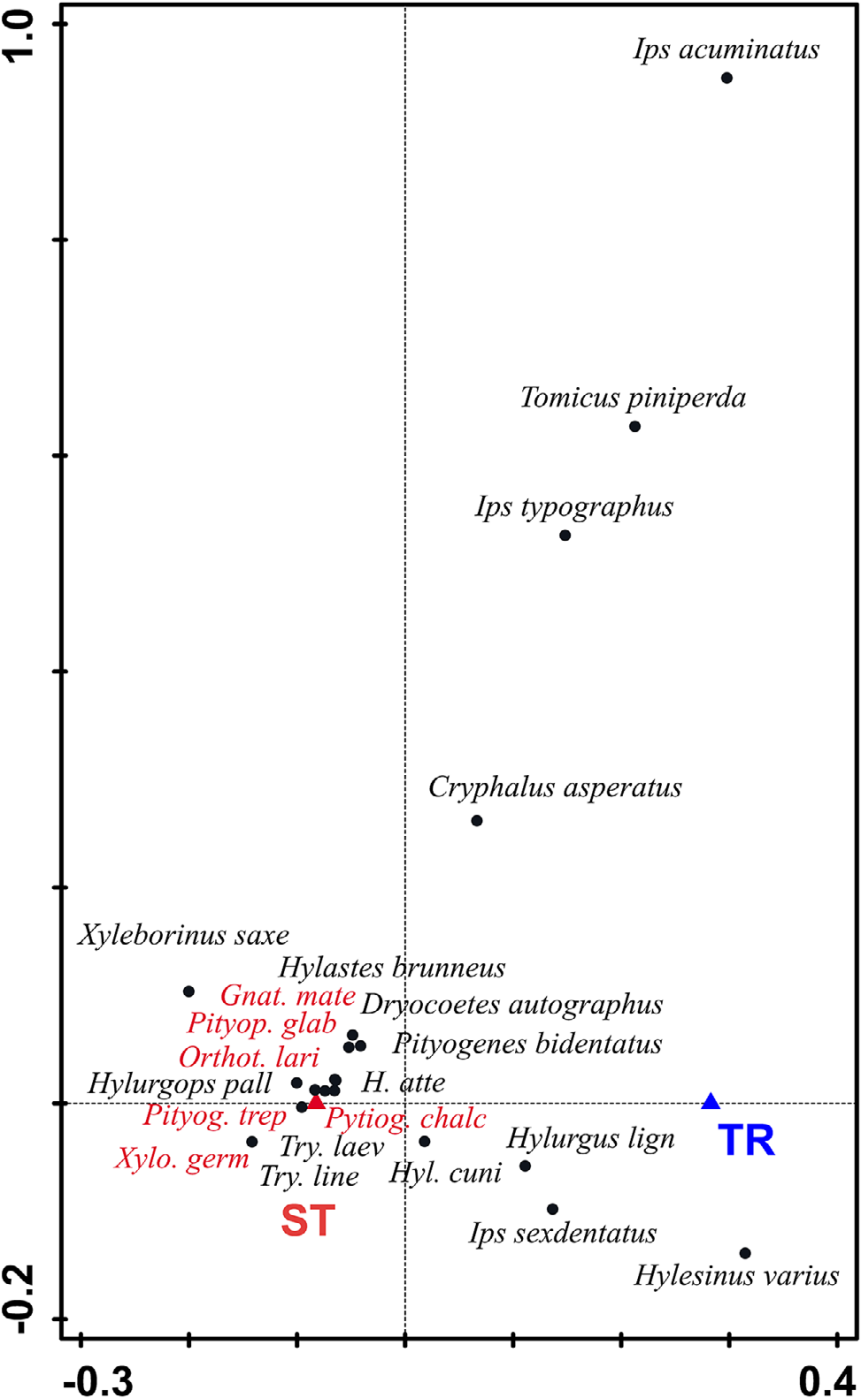
pCCA analysis plot of bark beetle species composition in relation to the trapping method. Species with red labels are significant (*p* < 0.05) indicator species for slot traps. TR, tripod; ST, slot traps.

A total of 499 individuals of nontarget bark beetles, representing 24 species, were captured during the study. The most abundant species were *Pityogenes chalcographus* and *Ips sexdentatus* Bör. Both methods (STs and tripods) had a significant effect (pCCA: *pseudo‐F* = 5.9, *P* = 0.011) on bark beetle species composition, and explained 11.3% of the observed variance in species, indicating that more bark beetle species were captured by STs. Six significant indicator species were found for STs (*p* < 0.05), but no indicator species were identified for tripods (Fig. 8).

## 4 DISCUSSION

The various trapping methods used for trapping *I. cembrae* have a number of adverse effects, and the most serious of which, the detrimental impact on nontarget invertebrates, was investigated in this study. Saproxylic beetles associated with conifers were the most heavily affected group, in particular predators, such as *T. formicarius*, killed in large numbers. Insecticide‐treated methods, such as tripods and baited trees, had the most detrimental impact on nontarget species. Several Red List species also were found to be killed in substantial numbers, especially the predatory darkling beetle *C. fraxini*. Considering the high mortality rates of *T. formicarius*, a key predator of bark beetles, resulting from the evaluated trapping methods, alongside their limited effectiveness in capturing the target bark beetle species, *I. cembrae*, the overall justification for these measures appears questionable.

### 4.1 Effects on nontarget saproxylic beetles

Saproxylic beetles, represented by 11 584 individuals, were the largest group of nontarget invertebrates captured in the present study, and almost all of them (98.4%) could be classified as associated with conifers. This result is not surprising, as the studied trapping methods were based on the usage of larch trees (or their parts) and bait lures used for trapping *I. cembrae*. Unsurprisingly, the majority of trapped species were associated with conifers, indicating that the trapping methods have negligible negative effects on species associated with broadleaved tree species.

Dividing the nontarget saproxylic beetles into groups based on their larval feeding habits revealed finer patterns. Predators were the most impacted group, primarily owing to the high numbers of *T. formicarius* individuals trapped. *Thanasimus formicarius* is a widespread predator of bark beetles.^47^ The high numbers can be explained by the kairomone response of *T. formicarius* to ipsenol and ipsdienol,^48^ which are components of the pheromone lure used in the traps.^49^ Predators were predominantly trapped by tripods and baited trees, whereas logs and nonbaited trees trapped far fewer individuals. These results are similar to those of Kula *et al*.,^41^ where, in a study targeting *I. typographus*, tripods killed more *Thanasimus* individuals than logs. Overall, nontarget phloeoxylophagous and saproxylophagous species also were captured in substantial numbers. Saproxylophagous species were most likely to be attracted to the logs and tripods by semiochemicals emitted as a result of their cutting.^50^ Tripods exhibited the highest captures of phloeoxylophagous species, primarily represented by bark beetles. Low numbers of mycetophagous species were trapped as they are usually associated with more decayed and humid microhabitats releasing substantially different odours, and were clearly not attracted to the traps.^51^ The most numerous Red List species trapped was *C. fraxini*. Darkling beetles of the genus *Corticeus* are bark beetles predators associated with coniferous species. Thus, it is not surprising that they were trapped in such high numbers.^52^

### 4.2 Effects on nontarget bark beetles

A wide range of nontarget bark beetles also were captured using the four trapping methods. Interactions between bark beetles and their hosts are based on semiochemicals.^21^ The response of an individual bark beetle species depends on the specific chiral structure of the chemical substances emitted from the host.^53^, ^54^ The Cembräwit pheromone lure used in this study contains ipsenol, ipsdienol, methyl butenol and amitinol.^49^ The capture of 140 individuals of *I. sexdentatus* can be explained by the lure's content of ipsdienol and ipsenol, which are part of the *I. sexdentatus* pheromone blend.^55^ Ipsdienol also has been detected in the hindgut of *P. chalcographus*, the most abundant bark beetle species trapped in this study, although it is not a major component of its pheromone blend.^56^ Closer inspection of the data revealed that 89% of the *P. chalcographus* individuals were captured at one site, indicating that the capture was probably caused by an outbreak of *P. chalcographus* in the vicinity of the site. The capture of *I. acuminatus*, albeit only one individual, can be explained by the presence of amitinol in the lure.^57^ Overall, our results indicate that the use of Cembräwit lures can be advantageous, as they allow trapping of *I. sexdentatus* and *P. chalcographus*, both of which are serious bark beetle forest pests, simultaneously with *I. cembrae*.^25^, ^58^

### 4.3 General consequences of using insecticide‐treated methods

During any spraying a certain amount of drift occurs. In this study, to mitigate spray drift, traps were sprayed only during suitable environmental conditions. Many factors can influence spray drift, such as wind speed, wind direction, air temperature, tank pressure, chemical application rate, nozzle design and droplet size.^59^ Although using insecticides is effective at killing pests, they often produce negative effects such as environmental pollution.^60^ This study focused on the negative effects of insecticides on terrestrial arthropods, yet cypermethrin, the most common insecticide and the one used in this study, is highly toxic to and dangerous for aquatic organisms (e.g. Osteichthyes, Trichoptera, Odonata).^61^, ^62^ Tree injection of systemic insecticides is an alternative to spraying and avoids spray drift.^63^ It has shown to be effective for the control of bark beetles,^64^ but is time‐consuming^65^ and difficult to implement in a large‐scale forestry setting.

Predators, with 7541 captured individuals, were the most affected group of nontarget invertebrates in the present study, which might have serious consequences for local saproxylic beetle communities, represented mainly by *T. formicarius* (97% of predator individuals) and also by Red List species of genus *Corticeus*. The mean longevity of a *T. formicarius* adult is 127 days and its bark beetle consumption varies from 1.5 to 4 individuals per day;^66^, ^67^ therefore, each *T. formicarius* adult is likely to consume many hundred bark beetle individuals during its lifespan. Furthermore, *T. formicarius* is highly fecund, with females producing an average of 100–200 eggs.^68^ Its larvae also are very voracious predators, each consuming an estimated 44 *I. typographus* larvae during its development.^69^ The 7289 *T. formicarius* individuals trapped in this study therefore have the potential to substantially reduce the bark beetle populations of the study sites, potentially by millions of individuals. This greatly exceeds the number of *I. cembrae* (119508), the target pest, trapped during the fieldwork.^12^, ^16^, ^17^ However, although the aforementioned numbers seem convincing, it is difficult to assess the real importance that *T. formicarius* has on bark beetle population dynamics in forest ecosystems. Weslien^70^ reported that populations of *T. formicarius* were 12‐fold bigger in outbreak areas than in uninfested stands. Many authors have stated that *T. formicarius* can dramatically reduce bark beetle populations.^5^, ^67^, ^71^ However, no published studies have directly investigated the effects of *T. formicarius* predation on bark beetle population dynamics, thus it is difficult to quantify its effect on recent European bark beetle outbreaks.^72^ Nonetheless, it is clear that the overall desired suppressive effect of the studied trapping methods on *I. cembrae* populations is questionable, they may even have the opposite effect, because they reduce numbers of *I. cembrae* predators.

In this study, we showed that insecticide‐treated trees and tripods captured more nontarget invertebrates than STs and logs. Although trees and logs were equally effective in trapping *I. cembrae*,^17^ logs captured fewer nontarget invertebrate individuals. This suggests that the use of logs for mass trapping of *I. cembrae* during outbreaks has fewer negative impacts on nontarget invertebrates while retaining efficacy against the target pest. Although STs captured similar numbers of nontarget invertebrates to the logs, their effectiveness in trapping *I. cembrae* is lower compared to the other methods.^12^, ^17^ No studies have been published on the overall effect of *I. cembrae* trapping methods. In our study, we used insecticides alpha‐cypermethrin and cypermethrin, which are now banned for plant protection in EU, but other pyrethroids such as deltamethrin, can be used as alternatives for protection against bark beetles. Based on current knowledge, baited and insecticide‐treated methods appear to have little effect on *I. typographus* population densities.^73^ Some authors have proved that salvage logging seems to be an effective alternative^18^, ^19^, ^73^ to insecticide‐treated methods to mitigate bark beetle outbreaks, with probably less negative effect on nontarget invertebrates.

## 5 CONCLUSION

Our results have shown that insecticide‐treated methods and STs for trapping *I. cembrae* have a significant negative effect on nontarget invertebrates across various taxa and feeding groups, and including Red List species. The negative effect on the predators may be sufficiently large enough to outweigh any benefits gained from a reduction in *I. cembrae* numbers resulting from trapping. Future studies assessing the impact of such predator reduction on bark beetle population dynamics in forest ecosystems are highly desirable. To reduce the negative impacts on nontarget invertebrates, properly timed and consistent salvage logging should be used rather than insecticide‐treated trapping methods.

## AUTHOR CONTRIBUTIONS

Jakub Špoula: conceptualization, data curation, formal analysis, investigation, methodology, software, visualization, writing – original draft. Jiří Foit: conceptualization, data curation, validation, writing – review & editing. Karolína Resnerová: data curation, writing – review & editing. Emanuel Kula: conceptualization, funding acquisition, investigation, methodology, resources, supervision, project administration, writing – review & editing. All authors have read and agreed to the published version of the manuscript.

## CONFLICT OF INTEREST

The authors have no conflict of interests.

## Data Availability

The data that support the findings of this study are available from the corresponding author upon reasonable request.
